# Gastric emptying rate and relevance for symptoms

**DOI:** 10.1002/ueg2.12379

**Published:** 2023-03-14

**Authors:** Hans Törnblom

**Affiliations:** ^1^ Department of Molecular and Clinical Medicine Institute of Medicine Sahlgrenska Academy University of Gothenburg Gothenburg Sweden

**Keywords:** gastroparesis, gastric empting rate, nausea, symptoms, vomiting

Nausea, vomiting, epigastric pain, early satiety, and fullness are common symptoms in the adult population. The outcome of symptom reports from the recent Rome Foundation Global Epidemiology Study^1^ showed that as many as 10.6% of adults in 26 countries fulfilled any of the diagnoses included among the gastroduodenal disorders,^2^ which by definition shows that these symptoms are present with chronicity. The number of subjects who have delayed gastric emptying time is unknown, and the proportion fulfilling the criteria for gastroparesis^3^ is therefore obscure. Anyhow, from the same Rome Foundation Global Epidemiology Study database, a specific symptom pattern was used as the definition of gastroparesis, such as nausea and/or vomiting ≥1 day/week and simultaneous postprandial distress syndrome, and this was reported by 0.9% of adults.^4^ The high prevalence of both the gastroduodenal and gastroparesis‐like symptom clusters and the associated effects they have on health outcomes, including increased healthcare consumption, indicate the need for effective treatment options to reduce symptoms. The recent United European Gastroenterology (UEG) and European Society for Neurogastroenterology and Motility consensus on gastroparesis^5^ that was based on a Delphi process of agreement on evidence‐based statements disappointingly showed that only the dopamine‐2 antagonists and 5‐HT4 agonists are recommended for usage. Even if not everyone agrees on this, it shows the magnitude of trouble doctors have when pharmacologically treating gastroparesis.

As being the defining abnormality of gastroparesis, delayed gastric emptying is believed to be of relevance for symptom generation where the intuitive positive effect of a prokinetic medication seems reasonable. What we also know is that the pathophysiology of gastroparesis is complex and includes mechanisms, such as impaired gastric accommodation, gastric sensory dysfunction, antroduodenal coordination abnormalities, electrical dysrhythmias, and pyloric dysfunction, all of which might contribute to the seemingly random observations of symptom intensity in relation to the gastric emptying status.^6^, ^7^ In the current issue of UEG journal, Goelen et al present a very useful meta‐analysis, which aim to update the knowledge on the association between prokinetic‐induced acceleration of gastric emptying time and symptom improvement.^8^ There are two often cited studies on the topic with conflicting results on this matter,^9^, ^10^ and in the current meta‐analysis, both methodologies from these are used and compared, only including studies with data that were sufficient for both analytical approaches. In brief, the results differ if considering improvement in symptoms from baseline or change in symptoms over placebo. Importantly, if the change over placebo is considered, there is a positive association between gastric emptying rate and symptom improvement. Thus, the authors stress the importance of placebo‐controlled trials and optimal gastric emptying test methodology for future trials to better determine the effects of accelerated gastric emptying and its effect on symptoms.

This study will be appreciated by many since it highlights the importance of stringent methodology and definitions, a matter where scientific controversies still contribute to specific problems in gastroparesis.^11^ Among the basic problems involved that are also mentioned but too often ignored when designing studies is how symptoms are registered when being a primary outcome parameter. With modern technology, such as the use of smart phone apps and random symptom registrations,^12^ increasing opportunities to have a better quality of symptom data by using devices that also provide a better temporal relationship between symptoms and physiological outcome parameters might be useful.

## CONFLICTS OF INTEREST STATEMENT

HT has served as an advisory board member/consultant and/or speaker for Biocodex, Takeda, Tillotts, VIPUN Medical, and Cinclus Pharma.

## Data Availability

Data sharing is not applicable to this article as no new data were created or analyzed in this study.
